# Supplementary material for: Single-cell transcriptomics of human T cells reveals tissue and activation signatures in health and disease
Source: Nat Commun. 2019 Oct 17;10:4706. doi: 10.1038/s41467-019-12464-3 (PMC6797728; doi:10.1038/s41467-019-12464-3)
Supplement: Supplementary file 1 — Supplementary Information [file 41467_2019_12464_MOESM1_ESM.pdf]

Supplementary Figures and Tables

Single-cell transcriptomics of human T cells reveals tissue and activation signatures in health and disease

Peter A. Szabo[†1], Hanna Mendes Levitin[†2], Michelle Miron[1,3], Mark E. Snyder[1], Takashi Senda[1,4], Jinzhou Yuan[2], Yim Ling Cheng[2], Erin C. Bush[2], Pranay Dogra[1], Puspa Thapa[1], Donna L. Farber[1,3,4‡*], and Peter A. Sims[2,5‡*]

[1] Columbia Center for Translational Immunology, Columbia University Irving Medical Center, New York, NY.

[2] Department of Systems Biology, Columbia University Irving Medical Center, New York, NY.

[3] Department of Microbiology and Immunology, Columbia University Irving Medical Center, New York, NY.

[4] Department of Surgery, Columbia University Irving Medical Center, New York, NY.

[5] Department of Biochemistry and Molecular Biophysics, Columbia University Irving Medical Center, New York, NY

*Address correspondence to: D.L. Farber (df2396@cumc.columbia.edu) and P.A. Sims (pas2182@cumc.columbia.edu)

[†]These authors contributed equally

[‡]These authors jointly supervised this work

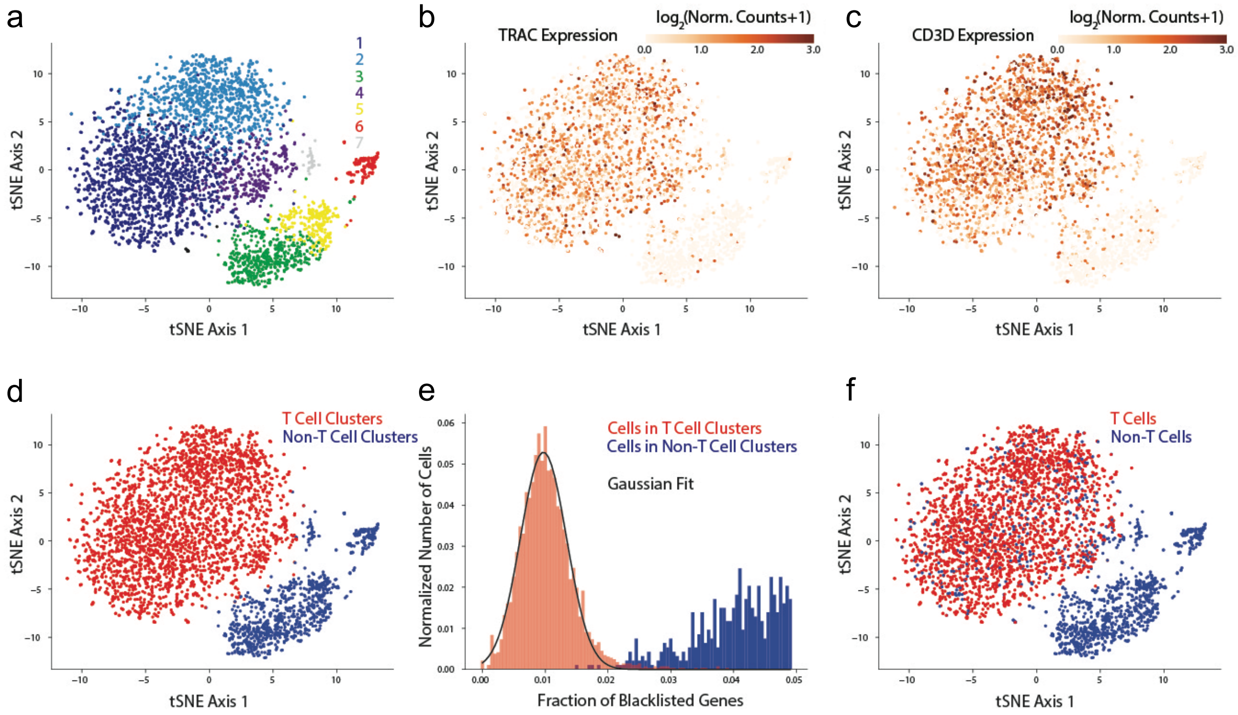

Supplementary Figure 1: Example of computational methods for identifying T cells from single-cell RNA-seq data. (a) tSNE projection scRNA-seq profiles of the resting cell sample from LG of Tissue Donor 1. Individual cells are colored by cluster membership identified using Phenograph (see Methods). (b) Same as (a) with cells colored by expression of TRAC, a highly expressed marker of T cells. (c) Same as (a) with cells colored by expression of *CD3D*, a highly expressed marker of T cells. (d) Same as (a) with cells colored by whether they are members of clusters that are enriched in *CD3D* expression (red) or not (blue). (e) Histograms of the fraction of blacklisted genes detected per cell for cells in clusters that are enriched in *CD3D* expression (red) and cells that are not (blue). The blacklisted genes are identified by differential expression analysis between the red and blue cells across the entire data set (see Methods). The black line is a Gaussian fit to the red histogram. (f) Same as (a) with cells colored by whether they are identified as T cells (red) or not (blue). All cells in clusters that are not enriched in *CD3D* are considered non-T cells. Cells in the clusters that are enriched in *CD3D* are considered non-T cells if the fraction of blacklisted genes detected exceeds two standard deviations above the mean of the Gaussian fit in (e).

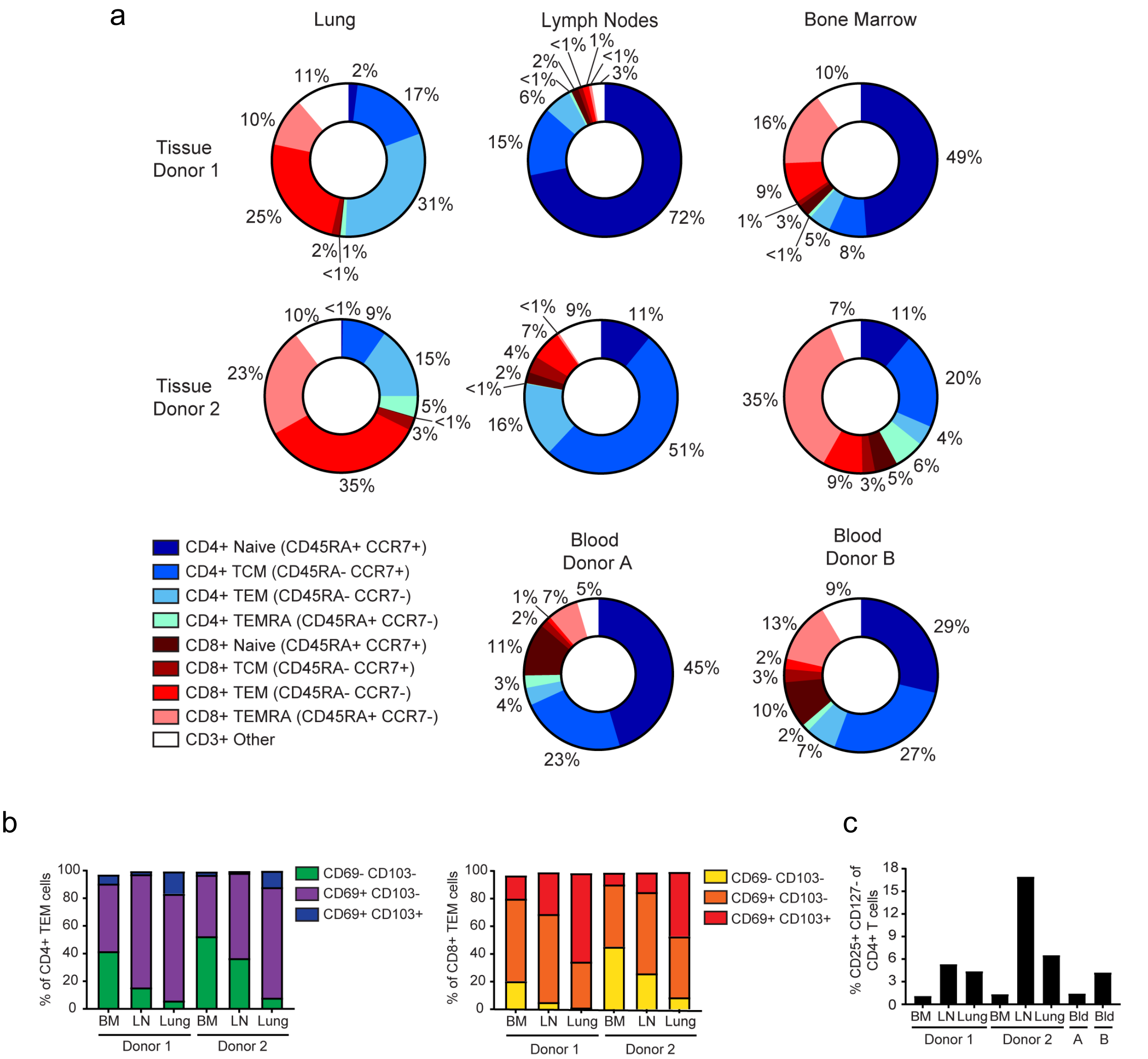

Supplementary Figure 2: T cell subset phenotypes for tissue and blood donors. (a) T cell subset composition of tissue and blood donors, showing frequency of naïve (CD45RA+ CCR7+), central-memory (TCM; CD45RA- CCR7+) effector-memory (TEM; CD45RA- CCR7-), and terminal effector (TEMRA; CD45RA+ CCR7-) subsets for $CD4^+$ and $CD8^+$ T cells. (b) Fraction of $CD4^+$ or CD8 TEM cells expressing tissue resident memory (TRM) markers CD69 and CD103 from the two tissue donors. (c) Fraction of $CD4^+$ T cells in each sample (both tissue and blood donors) that express a regulatory T cell (Treg) phenotype (CD25+CD127-).

a Blood Donor A on Tissue Donor 1

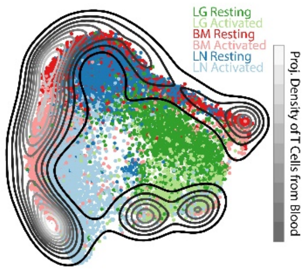

b

Resting T Cells from Blood

	LG	BM	LN
CD4 Rest.	105	2015	591
CD4 Act.	45	561	57
CD8 Rest.	3	277	343
CD8 Act.	4	280	1

Activated T Cells from Blood

	LG	BM	LN
CD4 Rest.	73	160	49
CD4 Act.	681	1290	1768
CD8 Rest.	6	13	39
CD8 Act.	84	185	563

c Blood Donor B on Tissue Donor 1

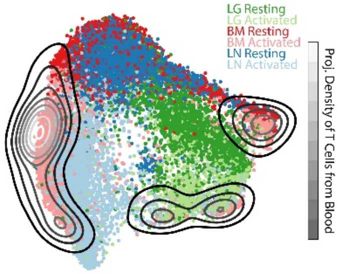

d

Resting T Cells from Blood

	LG	BM	LN
CD4 Rest.	196	492	124
CD4 Act.	161	1878	153
CD8 Rest.	29	225	130
CD8 Act.	104	664	40

Activated T Cells from Blood

	LG	BM	LN
CD4 Rest.	100	61	28
CD4 Act.	684	1053	1263
CD8 Rest.	19	55	35
CD8 Act.	93	260	585

e Blood Donor A on Tissue Donor 2

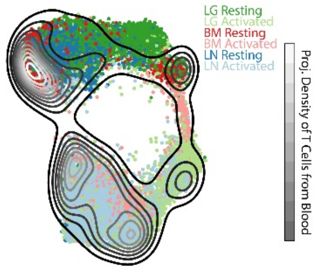

f

Resting T Cells from Blood

	LG	BM	LN
CD4 Rest.	397	2034	453
CD4 Act.	27	415	113
CD8 Rest.	18	521	294
CD8 Act.	1	1	8

Activated T Cells from Blood

	LG	BM	LN
CD4 Rest.	19	113	61
CD4 Act.	1088	1255	1438
CD8 Rest.	0	61	16
CD8 Act.	303	21	536

g Blood Donor B on Tissue Donor 2

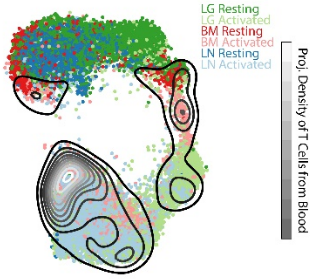

h

Resting T Cells from Blood

	LG	BM	LN
CD4 Rest.	57	390	240
CD4 Act.	41	1777	509
CD8 Rest.	8	687	150
CD8 Act.	173	69	95

Activated T Cells from Blood

	LG	BM	LN
CD4 Rest.	7	37	33
CD4 Act.	806	1077	1176
CD8 Rest.	2	113	14
CD8 Act.	378	76	517

Supplementary Figure 3: Projections of individual blood T cells onto UMAP embeddings of individual tissue T cells. (a) UMAP embedding of T cells from tissue donor 1 colored by tissue, overlaid by a contour plot corresponding to the projection of merged resting and activated T cells from blood donor A onto the tissue embedding. (b) Heatmaps showing the number of blood donor A T cells that project most closely to each tissue-activation combination in the tissue donor 1 UMAP embedding. (c) Same as (d) for blood donor B. (d) Same as (b) for blood donor B. (e) Same as (a) for tissue donor 2 and blood donor A. (f) Heatmaps showing the number of blood donor A T cells that project most closely to each tissue-activation combination in the tissue donor 2 UMAP embedding. (g) Same as (e) for blood donor B. (h) Same as (f) for blood donor B.

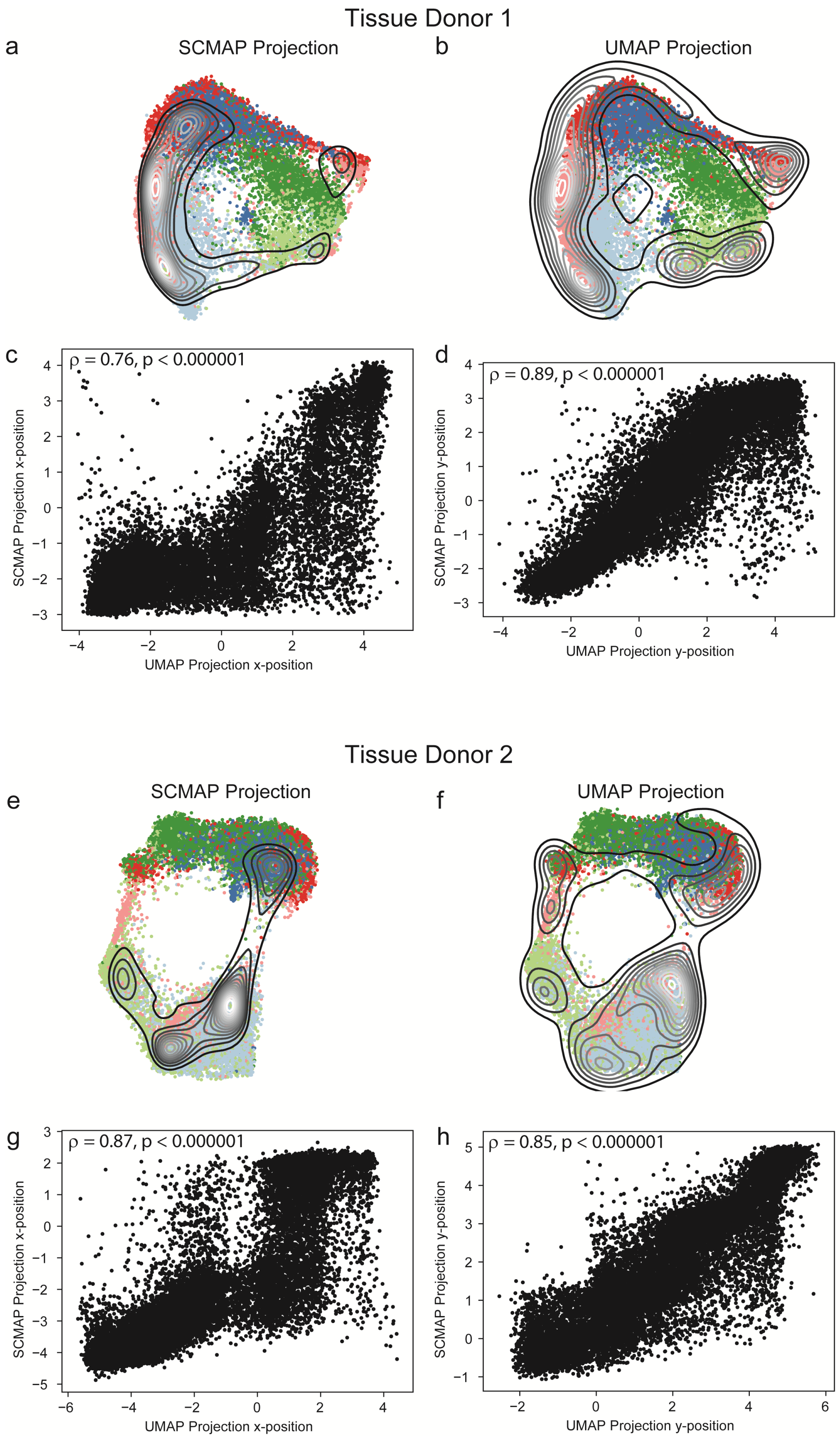
Tissue Donor 1
a
SCMAP Projection
b
UMAP Projection
c
ρ = 0.76, p < 0.000001
SCMAP Projection x-position
UMAP Projection x-position
d
ρ = 0.89, p < 0.000001
SCMAP Projection y-position
UMAP Projection y-position
Tissue Donor 2
e
SCMAP Projection
f
UMAP Projection
g
ρ = 0.87, p < 0.000001
SCMAP Projection x-position
UMAP Projection x-position
h
ρ = 0.85, p < 0.000001
SCMAP Projection y-position
UMAP Projection y-position

Supplementary Figure 4: Comparing UMAP and SCMAP for the projection of blood T cells onto tissue T cells for each donor. (a) Blood T cells from two donors projected onto tissue T cells from Tissue Donor 1 using SCMAP or (b) UMAP. (c, d) Coordinates for individual cells in UMAP and SCMAP projections. (e-h) Same as (a-d) but for Tissue Donor 2.

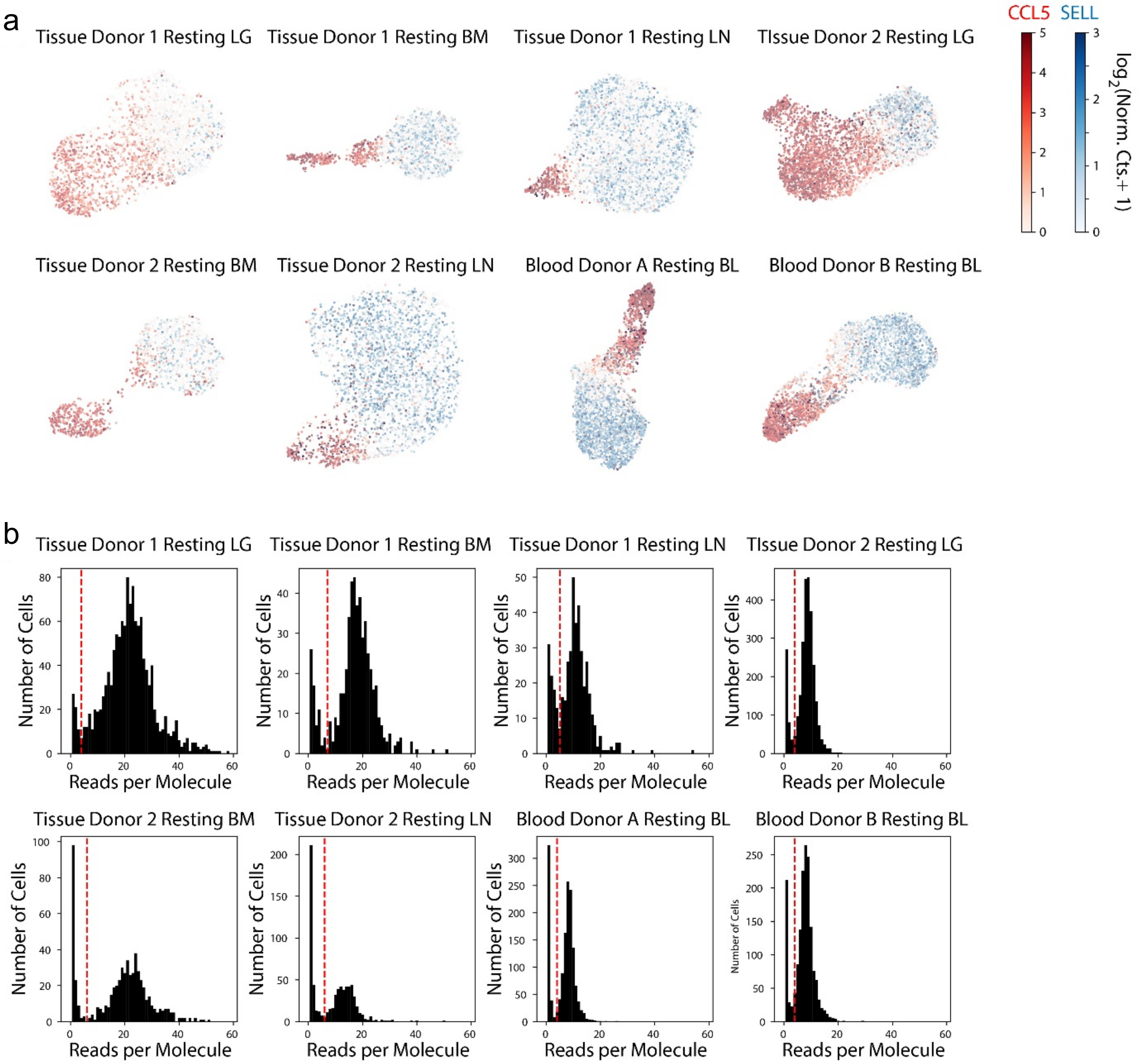

Supplementary Figure 5: Analysis of CCL5^{+} effector memory T cells. (a) UMAP embeddings of the resting T cells from each sample from each tissue and blood donor where each cell is colored by expression of *CCL5* (blue color bar) and *SELL* (red color bar). The two genes, which mark effector memory and non-effector memory populations, respectively, are essentially mutually exclusive. (b) Histograms of the average number of reads per molecule across cells in each sample from each tissue and blood onor for *CCL5*. The distributions are universally bimodal with the lower mode likely arising from molecular cross-talk. The dashed red line indicates the threshold below which the detection of *CCL5* was considered to be artifactual (see Methods) for each sample.

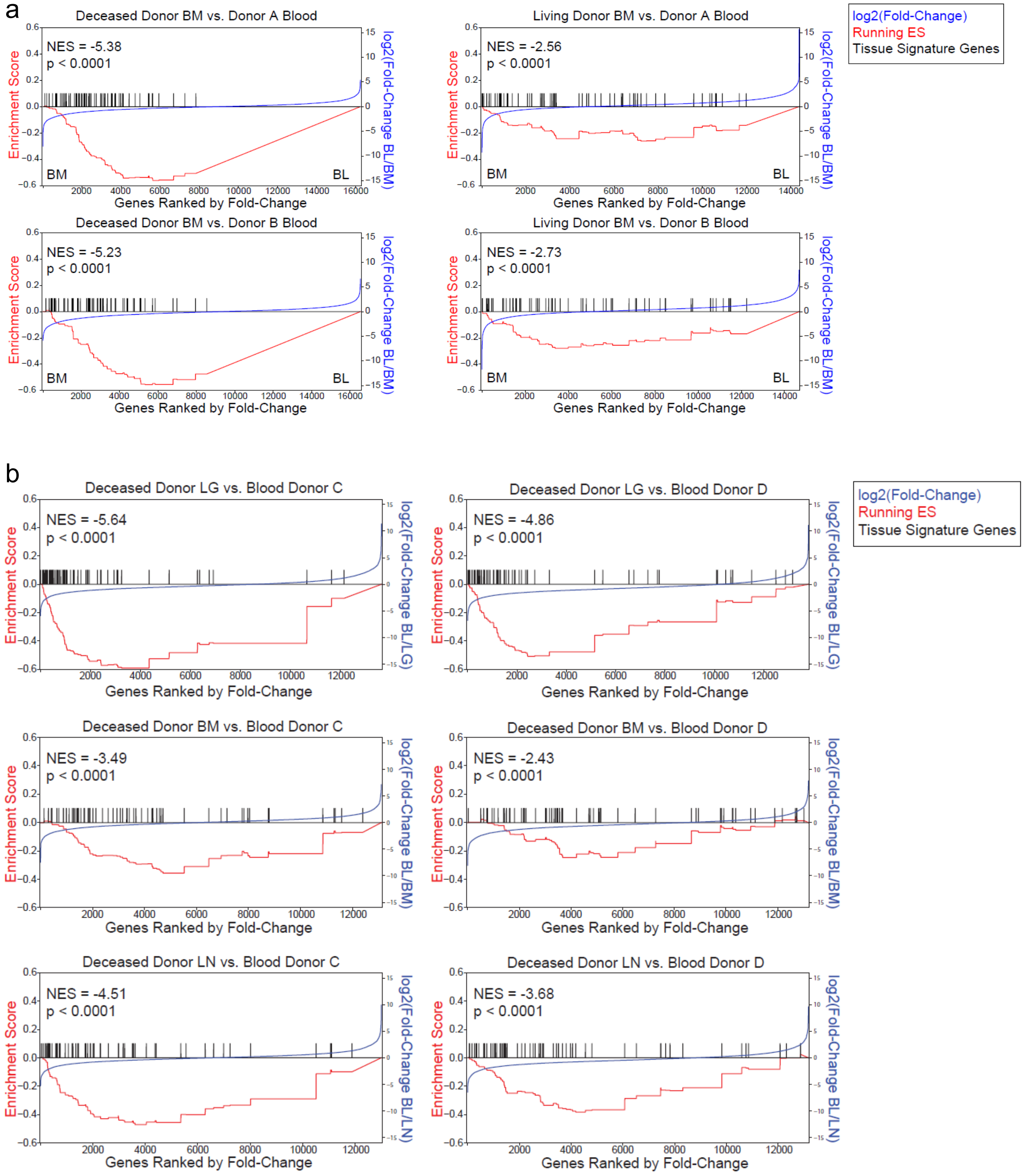

Supplementary Figure 6: Analysis of tissue-associated T cell signature genes in additional datasets. (a) Gene Set Enrichment Analysis (GSEA) comparing tissue-associated T cell signature genes in T cells from deceased (left) and living (right) BM donors relative to healthy living blood donors in our study. (b) GSEA comparing enrichment of T cell signature genes from deceased donor tissues (LG, BM, LN) to additional reference blood T cell datasets obtained from 10X Genomics (see Methods).

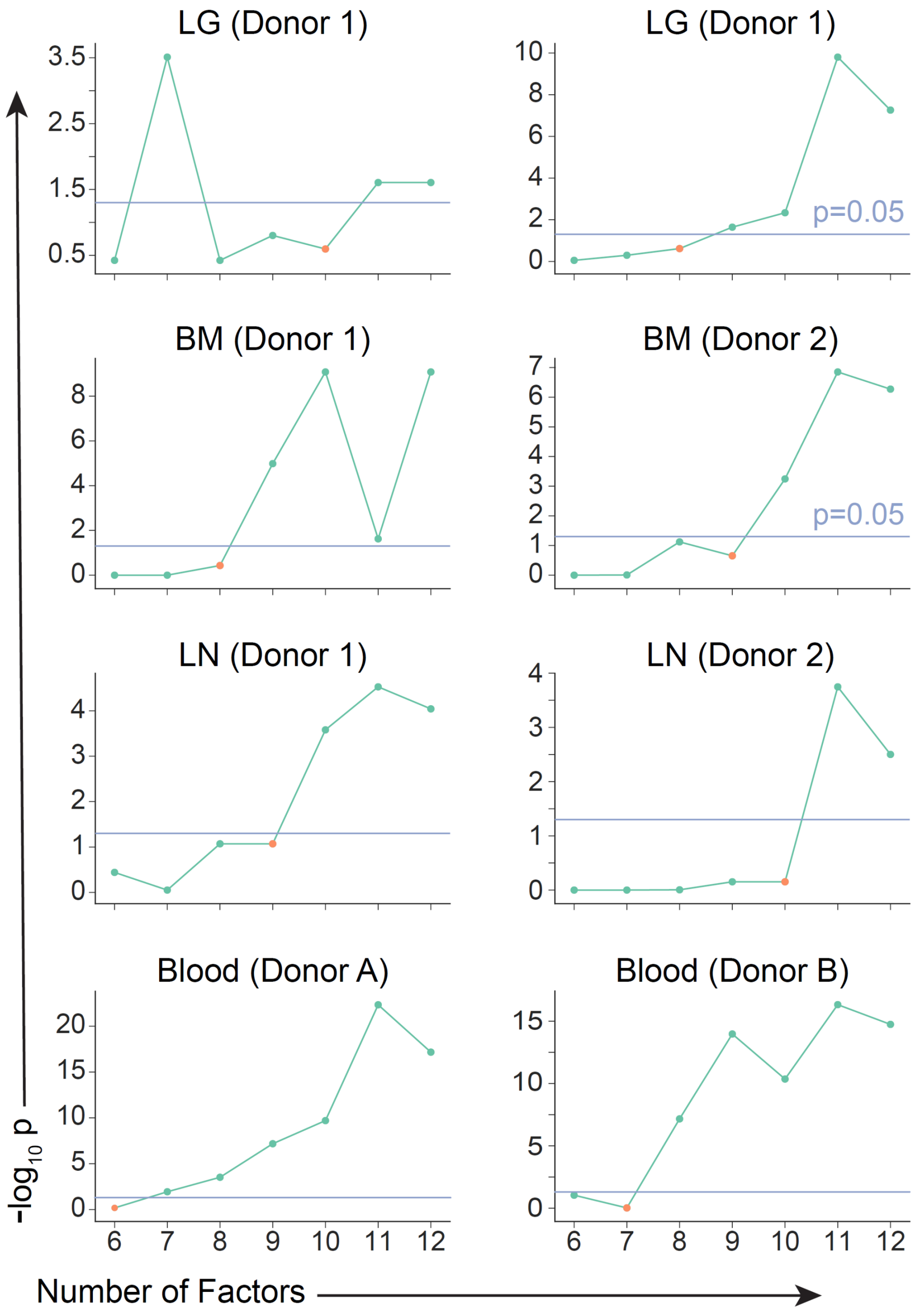

Supplementary Figure 7: Selection of the number of factors, *K*, for scHPF analysis of each sample. K was chosen to be the lowest value for which the p-value for pairwise overlap between the top 300 genes in each factor was less than 0.05 based on the hypergeometric test (see Methods). For each sample, the selected value of *K* is indicated by a orange dot.

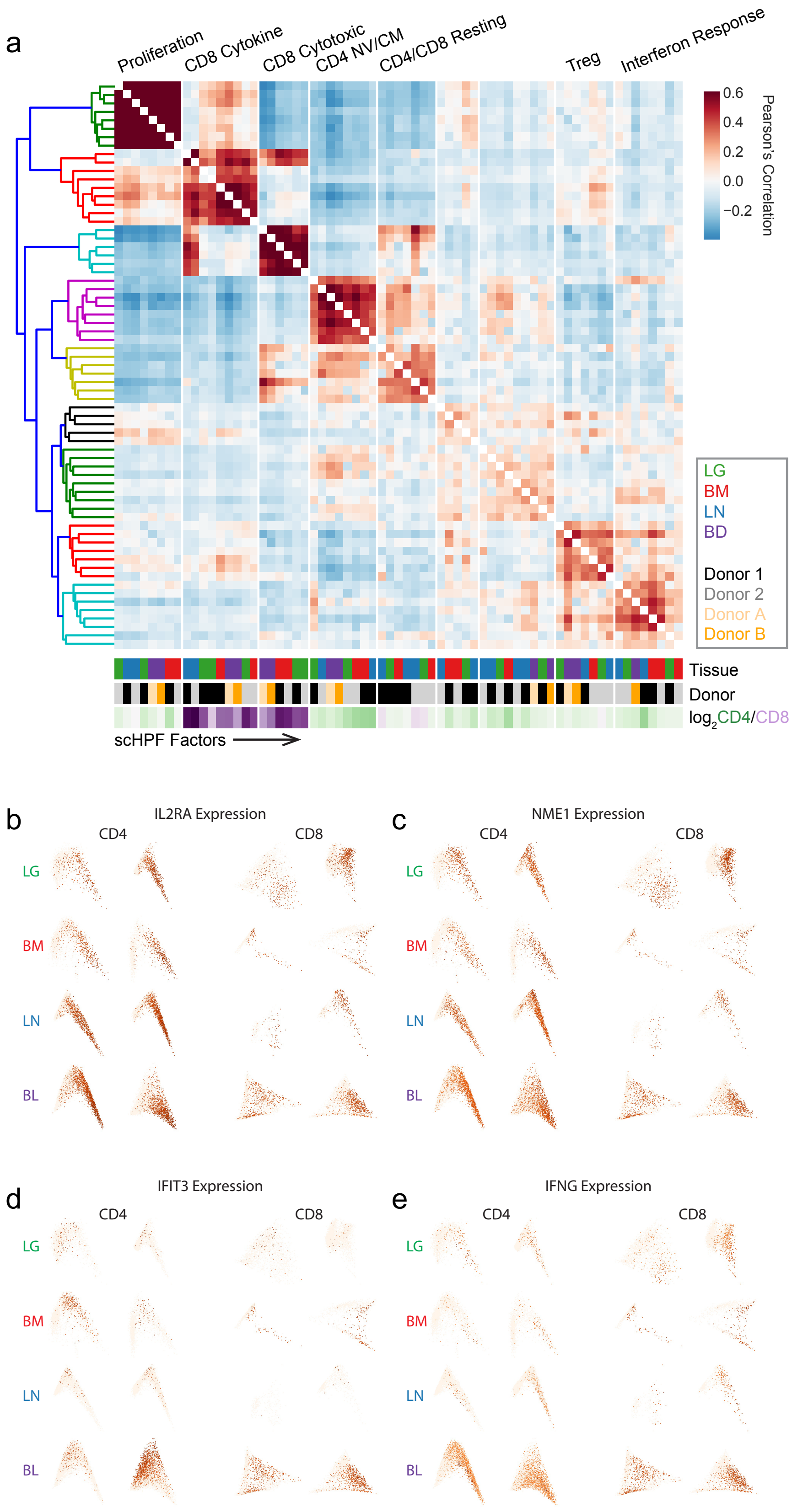

a
Proliferation
CD8 Cytokine
CD8 Cytotoxic
CD4 NV/CM
CD4/CD8 Resting
Treg
Interferon Response
0.6
0.4
0.2
0.0
−0.2
Pearson's Correlation
LG
BM
LN
BD
Donor 1
Donor 2
Donor A
Donor B
Tissue
Donor
log2CD4/CD8
scHPF Factors
b
IL2RA Expression
CD4
CD8
LG
BM
LN
BL
c
NME1 Expression
CD4
CD8
LG
BM
LN
BL
d
IFIT3 Expression
CD4
CD8
LG
BM
LN
BL
e
IFNG Expression
CD4
CD8
LG
BM
LN
BL

Supplementary Figure 8: scHPF clustergram and diffusion maps for select genes. (a) scHPF was used to factorize scRNA-seq profiles of each tissue and blood sample independently after merging resting and activated T cells. Each matrix element in the symmetrical clustergram is the pairwise Pearson's correlation coefficient between the gene scores for a pair of factors computed across a set of high- and low-scoring genes (see Methods). The resulting modules were named based on the identities of their highest scoring genes (Supplementary Data 5) and the resting vs. activated status of the highest scoring cells. Lower color bars show the tissue and donor of origin, and the CD4/CD8 bias of cell scores for each factor. (b) Diffusion maps generated from scHPF factors (see Methods) for $CD4^+$ and $CD8^+$ T cells from each tissue and blood sample after merging resting and activated T cells with each cell colored by expression of *IL2RA*. (c) Same as (b) but colored by expression of *NME1*. (d) Same as (b) but colored by expression of *IFIT3*. (e) Same as (b) but colored by expression of *IFNG*.

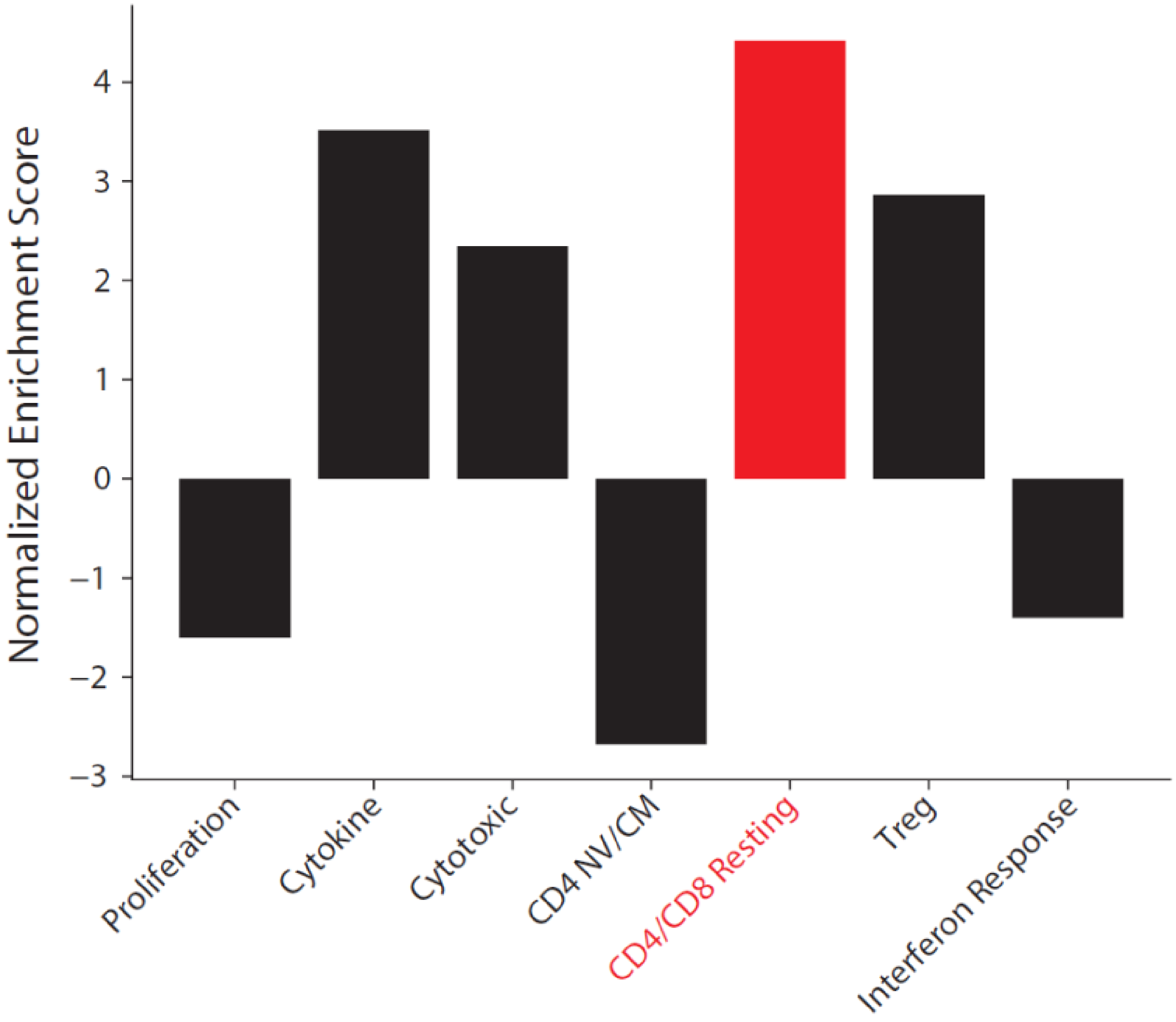

Supplementary Figure 9: Enrichment of tissue T cell signature in modules. We used gene set enrichment analysis (GSEA) to assess the enrichment of the tissue T cell signature from Fig. 3 in ranked gene lists for each module in Fig. 4 (genes ranked by scHPF gene score). The CD4/CD8 Resting module (red), which lacks factor from the blood, exhibited the highest enrichment ($p < 0.00001$) of any module.

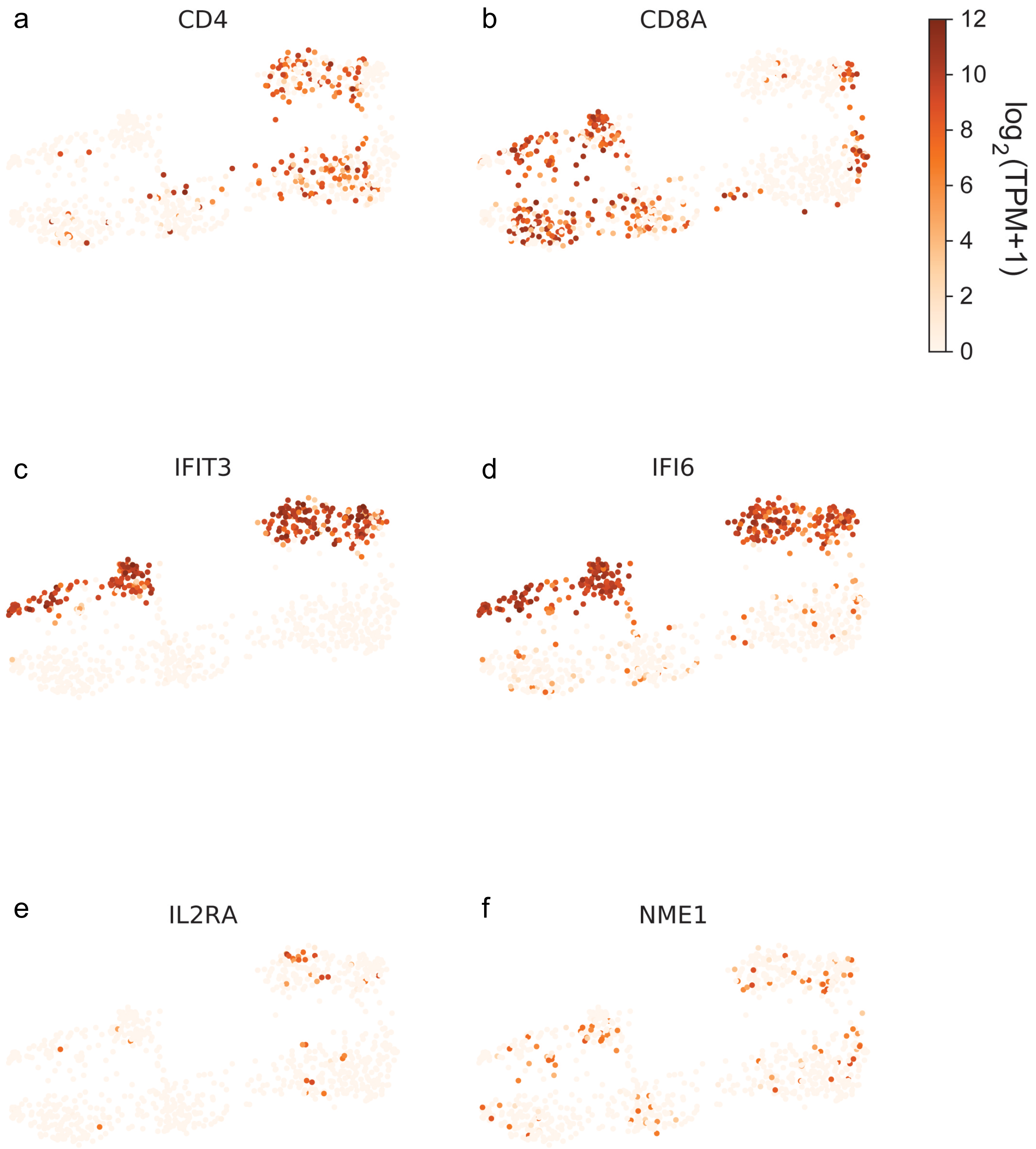

Supplementary Figure 10: UMAP embedding of PBMC-derived T cells from patients infected with dengue virus (data obtained from Ref. 49, main text). UMAP projections are colored by expression of (a) *CD4*, (b) *CD8A*, (c) *IFIT3*, (d) *IFI6* (a highly ranked gene in the IFN response module, see Supplementary Data 5), (e) *IL2RA*, and (f) *NME1*.

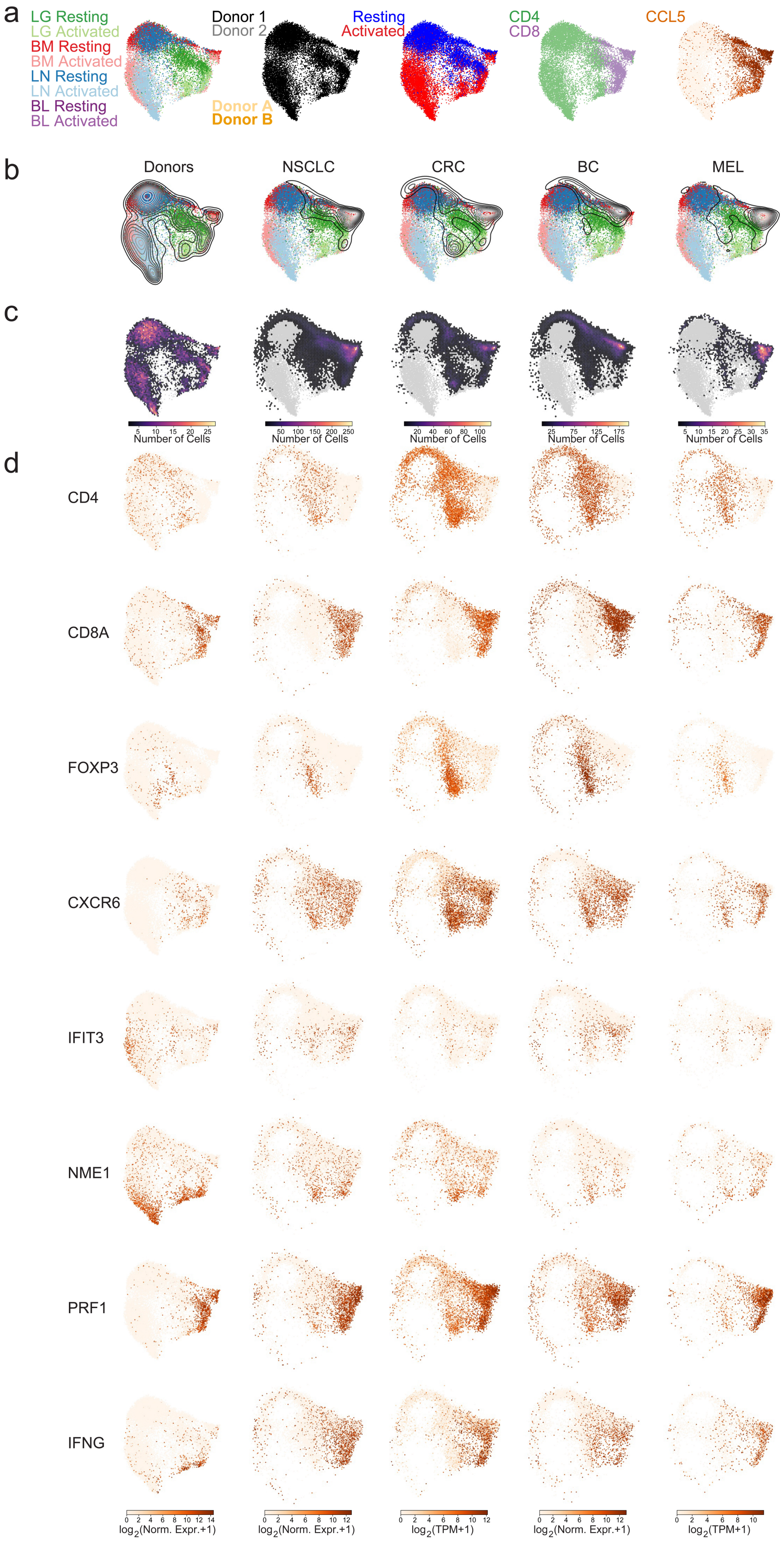
a
LG Resting
LG Activated
BM Resting
BM Activated
LN Resting
LN Activated
BL Resting
BL Activated
Donor 1
Donor 2
Donor A
Donor B
Resting
Activated
CD4
CD8
CCL5
b
Donors
NSCLC
CRC
BC
MEL
c
Number of Cells
d
CD4
CD8A
FOXP3
CXCR6
IFIT3
NME1
PRF1
IFNG
log2(Norm. Expr.+1)
log2(TPM+1)

Supplementary Figure 11: Comparison of tumor-associated T cells to a reference map of healthy human T activation generated exclusively from Donor 1 (includes LG, BM, and LN). (a) Merged UMAP embedding for Donor 1 including resting and activated T cells colored by sample source, donor, resting/activated condition, CD4/CD8 status, and *CCL5* expression indicating TEM cells. (b) Merged UMAP embedding for the entire dataset overlaid with contour plots indicating kernel density estimates for the projection of T cells derived from Donor 1 (column 1), non-small cell lung cancer tissue (columns 2), colorectal cancer (CRC) tissue (column 3), breast cancer (BC) tissue (column 4), and melanoma (MEL) tissue (column 5). Note that these probability densities can be compared within each projection, but cannot be quantitatively compared across projections. (c) Same as (b) but overlaid with a two-dimensional hexbin histogram for each projection. Histograms have been normalized to account for differences in cell numbers across datasets and therefore can be compared quantitatively across projections. (d) Individual cells in the UMAP embedding (column 1) for Donor 1 T cells and UMAP projections (columns 2-5) for NSCLC, CRC, BC, and MEL tissue T cells colored by expression of *CD4*, *CD8A, FOXP3* (Treg marker), *CXCRF6* (TRM marker), *IFIT3* (IFN response marker), *NME1* (activation marker), *PRF1* (cytotoxic marker), and *IFNG*. Expression values are normalized for quantitative comparison within each dataset (i.e. column), but not across datasets.

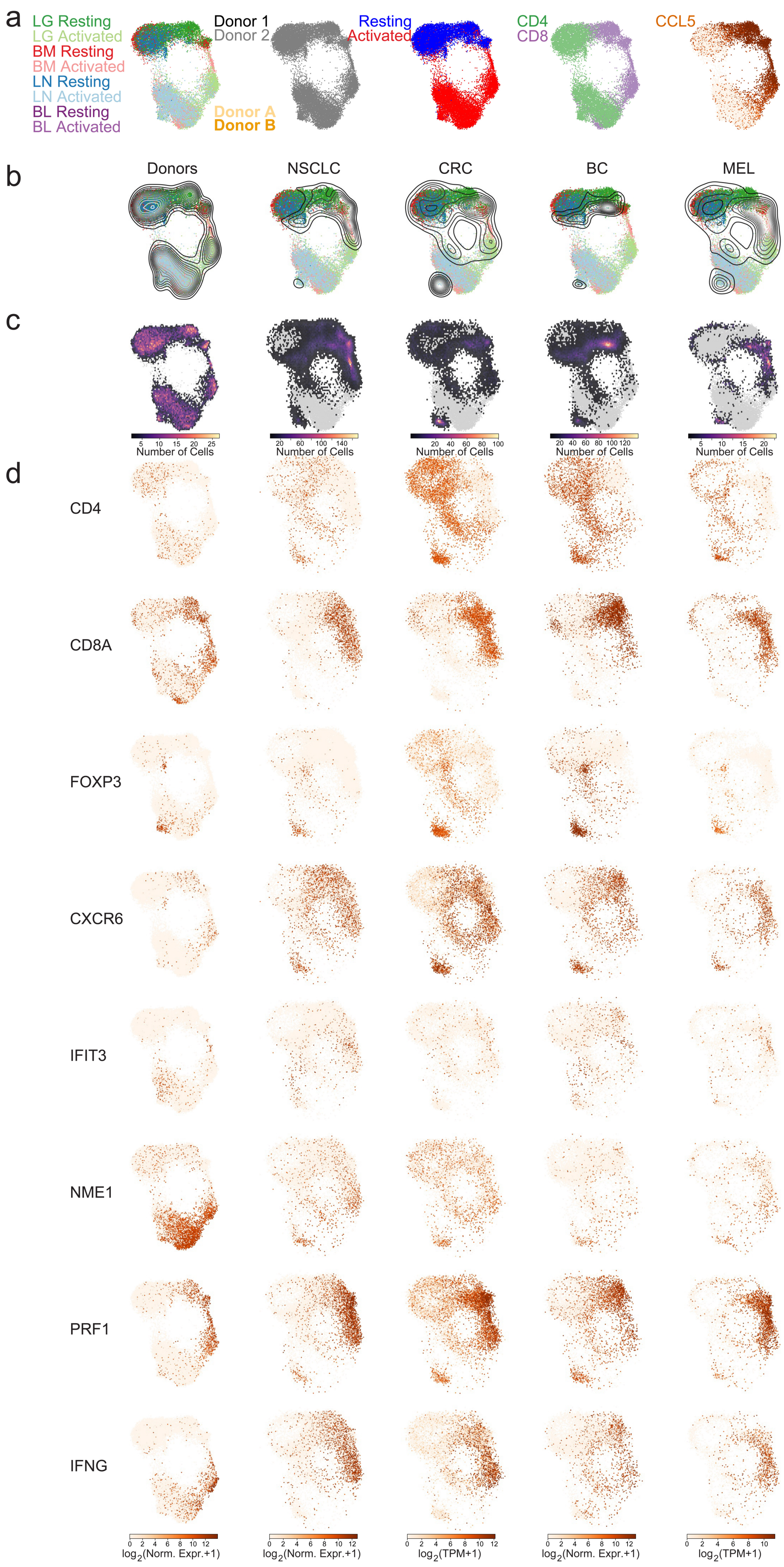
a
LG Resting
LG Activated
BM Resting
BM Activated
LN Resting
LN Activated
BL Resting
BL Activated
Donor A
Donor B
Donor 1
Donor 2
Resting
Activated
CD4
CD8
CCL5
b
Donors
NSCLC
CRC
BC
MEL
c
Number of Cells
d
CD4
CD8A
FOXP3
CXCR6
IFIT3
NME1
PRF1
IFNG
log2(Norm. Expr.+1)
log2(TPM+1)

Supplementary Figure 12: Comparison of tumor-associated T cells to a reference map of healthy human T activation generated exclusively from Donor 2 (includes LG, BM, and LN). (a) Merged UMAP embedding for Donor 2 including resting and activated T cells colored by sample source, donor, resting/activated condition, CD4/CD8 status, and *CCL5* expression indicating TEM cells. (b) Merged UMAP embedding for the entire dataset overlaid with contour plots indicating kernel density estimates for the projection of T cells derived from Donor 2 (column 1), non-small cell lung cancer tissue (columns 2), colorectal cancer (CRC) tissue (column 3), breast cancer (BC) tissue (column 4), and melanoma (MEL) tissue (column 5). Note that these probability densities can be compared within each projection, but cannot be quantitatively compared across projections. (c) Same as (b) but overlaid with a two-dimensional hexbin histogram for each projection. Histograms have been normalized to account for differences in cell numbers across datasets and therefore can be compared quantitatively across projections. (d) Individual cells in the UMAP embedding (column 1) for Donor 2 T cells and UMAP projections (columns 2-5) for NSCLC, CRC, BC, and MEL tissue T cells colored by expression of *CD4*, *CD8A, FOXP3* (Treg marker), *CXCRF6* (TRM marker), *IFIT3* (IFN response marker), *NME1* (activation marker), *PRF1* (cytotoxic marker), and *IFNG*. Expression values are normalized for quantitative comparison within each dataset (i.e. column), but not across datasets.

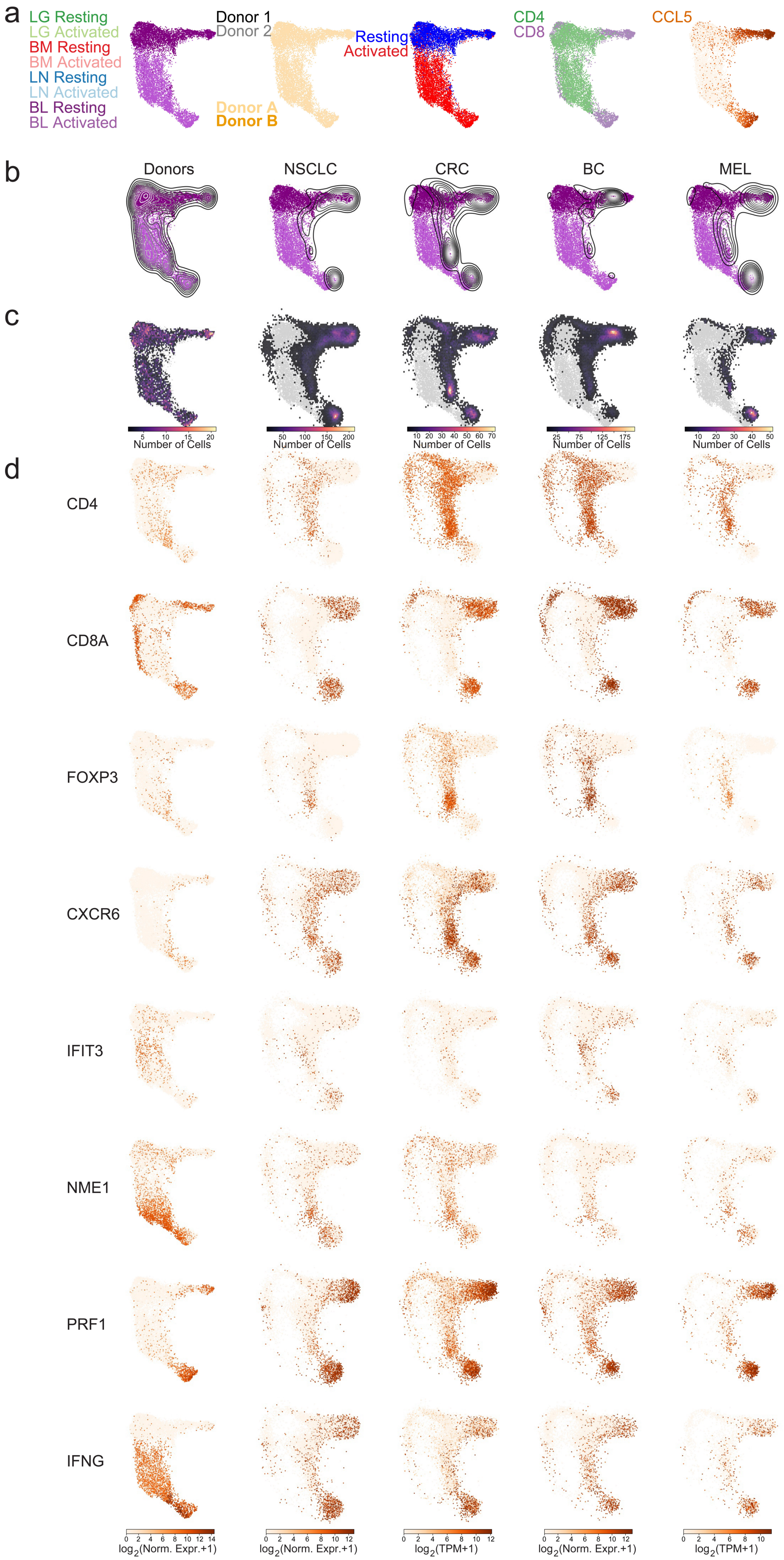
a
LG Resting
LG Activated
BM Resting
BM Activated
LN Resting
LN Activated
BL Resting
BL Activated
Donor 1
Donor 2
Donor A
Donor B
Resting
Activated
CD4
CD8
CCL5
b
Donors
NSCLC
CRC
BC
MEL
c
Number of Cells
d
CD4
CD8A
FOXP3
CXCR6
IFIT3
NME1
PRF1
IFNG
log2(Norm. Expr.+1)
log2(TPM+1)

Supplementary Figure 13: Comparison of tumor-associated T cells to a reference map of healthy human T activation generated exclusively from Donor A (includes blood only). (a) Merged UMAP embedding for Donor A including resting and activated T cells colored by sample source, donor, resting/activated condition, CD4/CD8 status, and *CCL5* expression indicating TEM cells. (b) Merged UMAP embedding for the entire dataset overlaid with contour plots indicating kernel density estimates for the projection of T cells derived from Donor A (column 1), non-small cell lung cancer tissue (columns 2), colorectal cancer (CRC) tissue (column 3), breast cancer (BC) tissue (column 4), and melanoma (MEL) tissue (column 5). Note that these probability densities can be compared within each projection, but cannot be quantitatively compared across projections. (c) Same as (b) but overlaid with a two-dimensional hexbin histogram for each projection. Histograms have been normalized to account for differences in cell numbers across datasets and therefore can be compared quantitatively across projections. (d) Individual cells in the UMAP embedding (column 1) for Donor A T cells and UMAP projections (columns 2-5) for NSCLC, CRC, BC, and MEL tissue T cells colored by expression of *CD4*, *CD8A, FOXP3* (Treg marker), *CXCRF6* (TRM marker), *IFIT3* (IFN response marker), *NME1* (activation marker), *PRF1* (cytotoxic marker), and *IFNG*. Expression values are normalized for quantitative comparison within each dataset (i.e. column), but not across datasets.

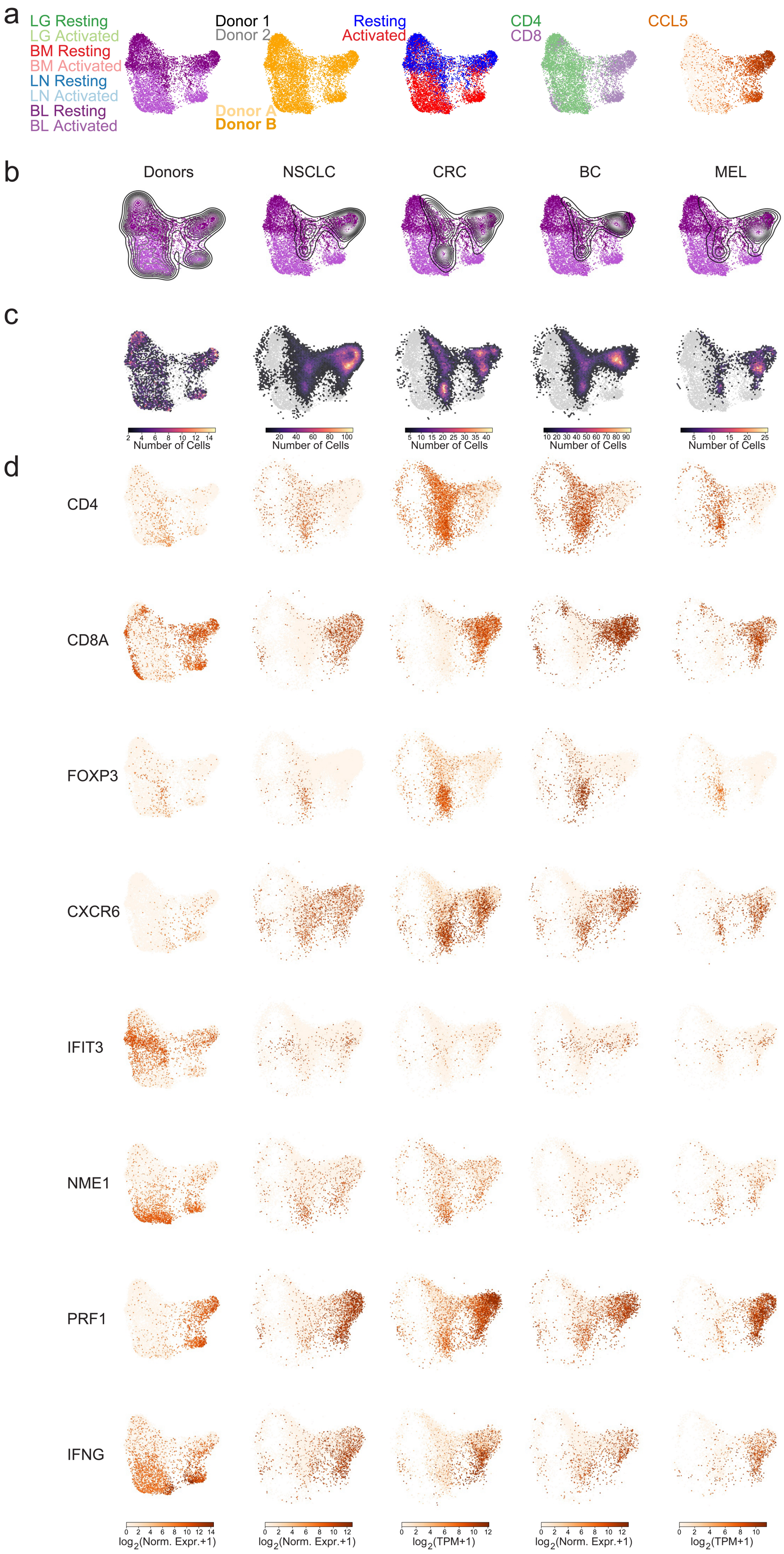
a
LG Resting
LG Activated
BM Resting
BM Activated
LN Resting
LN Activated
BL Resting
BL Activated
Donor 1
Donor 2
Donor A
Donor B
Resting
Activated
CD4
CD8
CCL5
b
Donors
NSCLC
CRC
BC
MEL
c
Number of Cells
Number of Cells
Number of Cells
Number of Cells
Number of Cells
d
CD4
CD8A
FOXP3
CXCR6
IFIT3
NME1
PRF1
IFNG
log2(Norm. Expr.+1)
log2(Norm. Expr.+1)
log2(TPM+1)
log2(Norm. Expr.+1)
log2(TPM+1)

Supplementary Figure 14: **Comparison of tumor-associated T cells to a reference map of healthy human T activation generated exclusively from Donor B (includes blood only).** (a) Merged UMAP embedding for Donor B including resting and activated T cells colored by sample source, donor, resting/activated condition, CD4/CD8 status, and *CCL5* expression indicating TEM cells. (b) Merged UMAP embedding for the entire dataset overlaid with contour plots indicating kernel density estimates for the projection of T cells derived from Donor B (column 1), non-small cell lung cancer tissue (columns 2), colorectal cancer (CRC) tissue (column 3), breast cancer (BC) tissue (column 4), and melanoma (MEL) tissue (column 5). Note that these probability densities can be compared within each projection, but cannot be quantitatively compared across projections. (c) Same as (b) but overlaid with a two-dimensional hexbin histogram for each projection. Histograms have been normalized to account for differences in cell numbers across datasets and therefore can be compared quantitatively across projections. (d) Individual cells in the UMAP embedding (column 1) for Donor B T cells and UMAP projections (columns 2-5) for NSCLC, CRC, BC, and MEL tissue T cells colored by expression of *CD4, CD8A, FOXP3* (Treg marker), *CXCRF6* (TRM marker), *IFIT3* (IFN response marker), *NME1* (activation marker), *PRF1* (cytotoxic marker), and *IFNG*. Expression values are normalized for quantitative comparison within each dataset (i.e. column), but not across datasets.

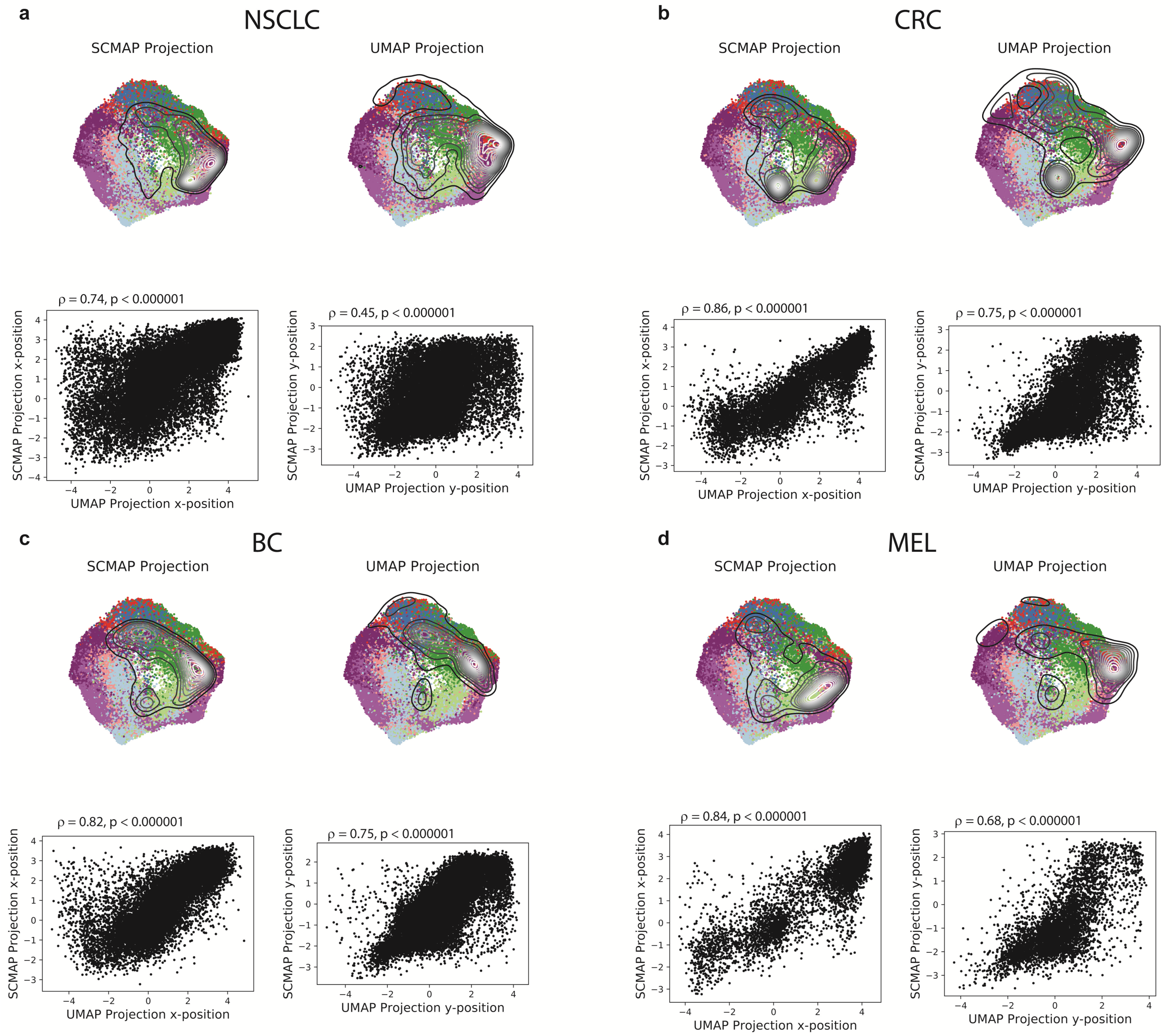

Supplementary Figure 15: Comparing UMAP and SCMAP for the projection of T cells from cancer onto the reference map. UMAP and SCMAP embeddings and cell projection coordinates for tumor-associated T cells from (a) non-small cell lung cancer tissue, (b) colorectal cancer tissue, (c) breast cancer tissue, or (d) melanoma tissue that are projected onto the reference map.

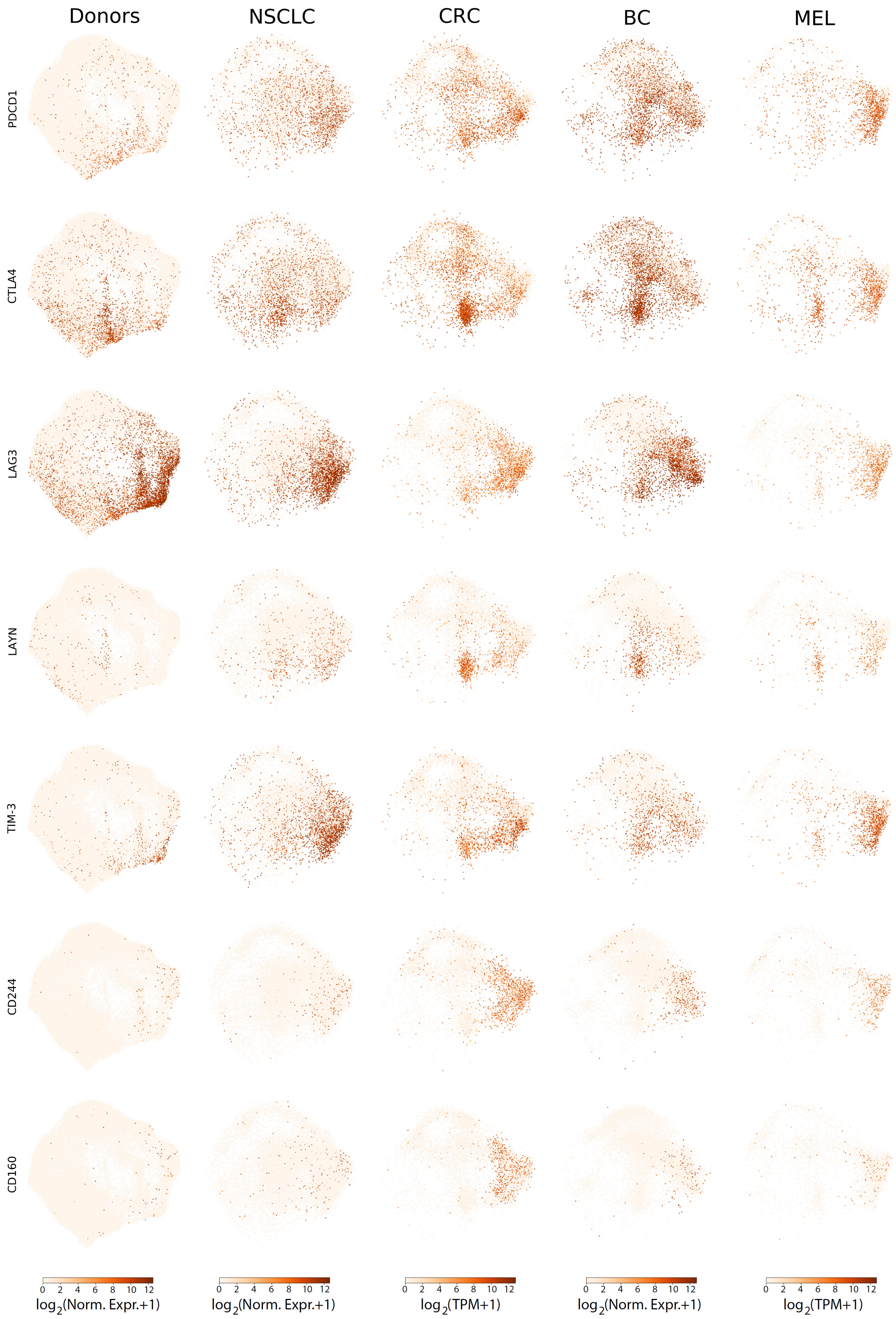
Donors
NSCLC
CRC
BC
MEL
PDCD1
CTLA4
LAG3
LAYN
TIM-3
CD244
CD160
0 2 4 6 8 10 12
log2(Norm. Expr.+1)
log2(Norm. Expr.+1)
log2(TPM+1)
log2(Norm. Expr.+1)
log2(TPM+1)

Supplementary Figure 16: Expression of exhaustion markers in organ and blood donor T cells and tumor-associated T cells. Using the UMAP embedding of the merged organ donor and blood T cells from Fig. 6 and the UMAP projections of the tumor-associated T cells from four tumor types onto this embedding, we colored individual cells in each dataset based on expression of T cell exhaustion markers.

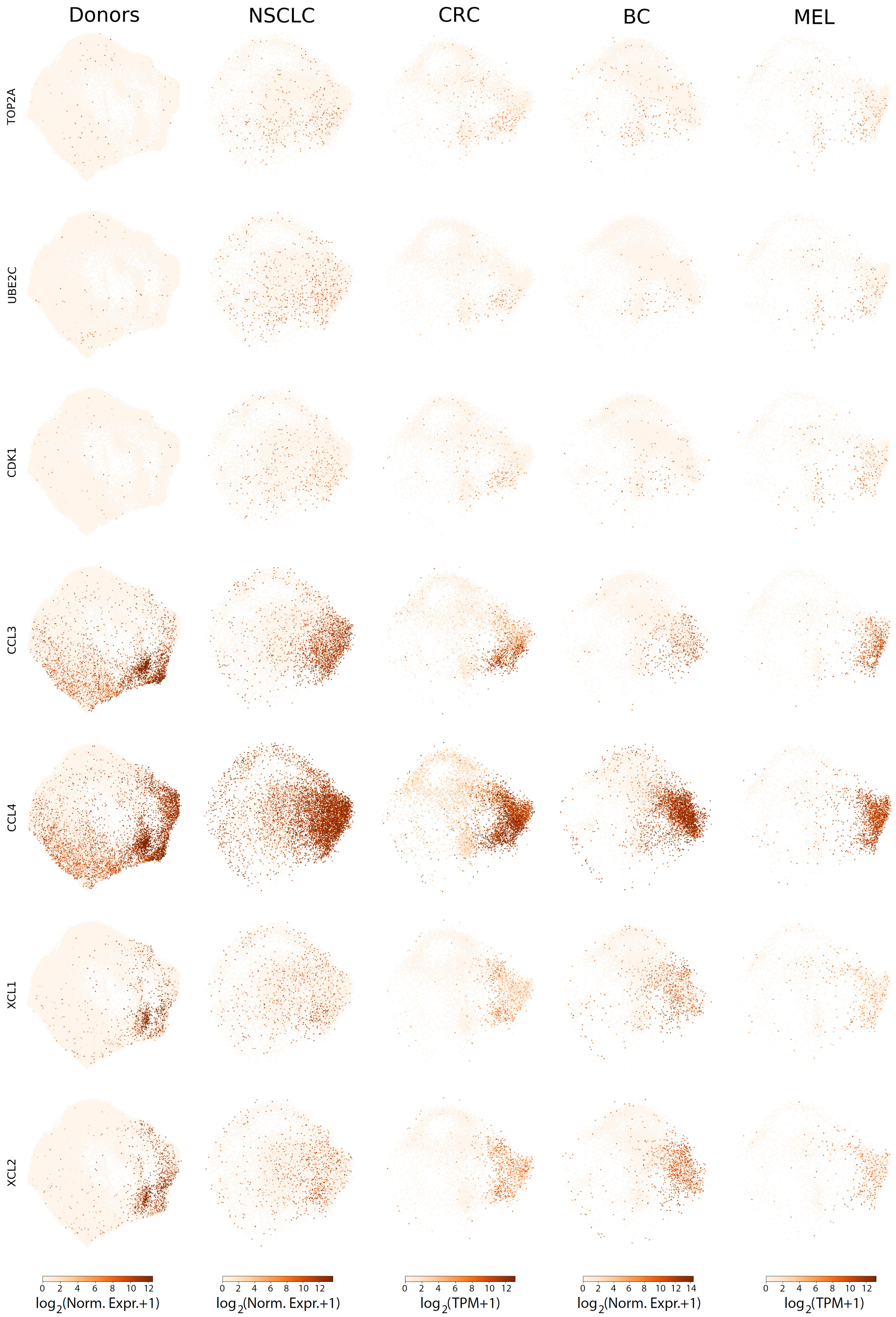

Donors
NSCLC
CRC
BC
MEL
TOP2A
UBE2C
CDK1
CCL3
CCL4
XCL1
XCL2
0 2 4 6 8 10 12
log2(Norm. Expr.+1)
0 2 4 6 8 10 12
log2(Norm. Expr.+1)
0 2 4 6 8 10 12
log2(TPM+1)
0 2 4 6 8 10 12 14
log2(Norm. Expr.+1)
0 2 4 6 8 10 12
log2(TPM+1)

Supplementary Figure 17: Expression of cell cycle and chemokine genes in organ and blood donor T cells and tumor-associated T cells. Using the UMAP embedding of the merged organ donor and blood T cells from Fig. 6 and the UMAP projections of the tumor-associated T cells from four tumor types onto this embedding, we colored individual cells in each dataset based on expression of cell cycle markers (*TOP2A*, *UBE2C, CDK1*) and chemokines (*CCL3*, *CCL4*, *XCL1*, *XCL2*).

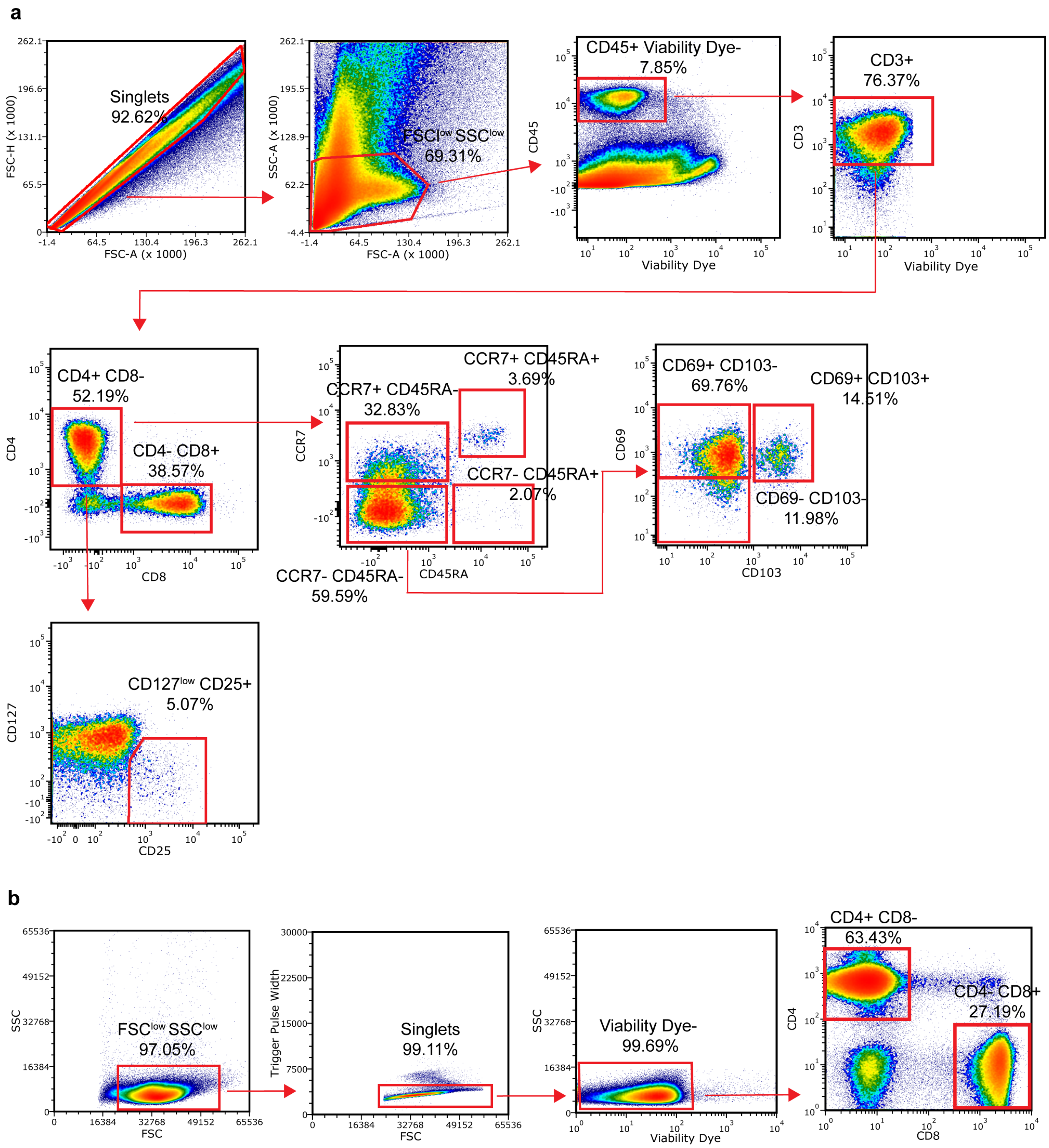

Supplementary Figure 18: Gating and sorting strategy for blood and tissue T cells. (a) Frequencies of $CD4^+$ T cell subsets isolated from the lungs of Tissue Donor 1 are shown as a representative gating strategy for phenotypic analysis of blood and tissue T cells in Supplementary Figure 2. All samples were first gated on singlets, FSC^{low}/SSC^{low}, $CD45^+$ and Viability Dye^- (indicating live cells) before gating for CD3 and subsequent lineage /subset markers. All samples from both tissue and blood Donors were analyzed similarly. Data was acquired on a BD LSRII cytometer and analyzed by FCS Express software. (b) Sorting strategy for blood $CD4^+$ and $CD8^+$ T cells for gene expression analysis in Fig 5a,c,d,e. Cells were magnetically enriched for $CD3^+$ T cells and pre-gated for FSC^{low}/SSC^{low}, singlets, and live cells for sorting.

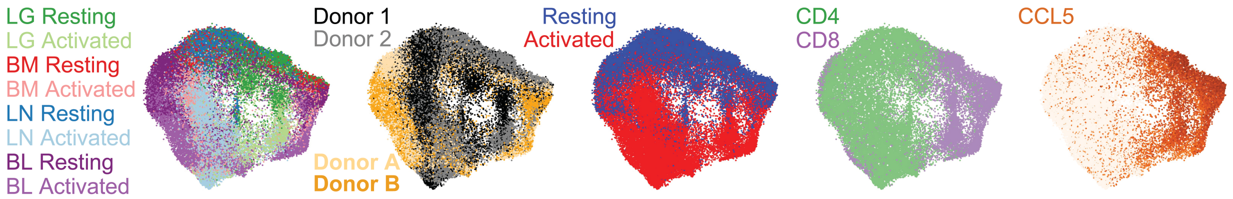

Supplementary Figure 19: Merged UMAP embedding for the entire human T cell scRNA-seq dataset including generated from a subset of the 315 highly variable genes that are not specific to any one donor. The embedding is colored by sample source, donor, resting/activated condition, CD4/CD8 status, and *CCL5* expression indicating TEM cells.

	Tissue Donor 1	Tissue Donor 2
**Demographics**		
Sex	Male	Male
Age (years)	65	52
Ethnicity/race	White	Hispanic/Latino
Body Mass Index (kg/m^2)	24.1	30.7
**Clinical Characteristics**		
Cause of Death	Cerebrovascular Accident/Stroke	Head Trauma
Mechanism of Injury	Intracranial Hemorrhage	Gunshot Wound
CPR administered	–	+
**Comorbidities**		
Hypertension	+	+
Diabetes	–	–
CAD	–	–
**Social History**		
Smoking History	–	+
Alcohol Use	+	+
I.V. Drug Use	–	–
**Serology**		
CMV	–	+
EBV	+	+
Toxoplasma	–	–

CAD, coronary artery disease; CMV, cytomegalovirus; CPR, cardiopulmonary resuscitation; EBV, Epstein Barr virus.

Supplementary Table 1: Demographic and clinical information from human organ donors.

	Tissue Donor 1	Tissue Donor 2
**Lung (Resting/Activated)**		
Total Cells	3,809 / 2,463	5,577 / 4,365
T cells	2,488 / 1,446	4,089 / 3,036
T cell fraction	0.653 / 0.587	0.710 / 0.696
Average Transcript Molecules Detected (T cells)	2,045 / 3,511	2,353 / 3,622
**Bone Marrow (Resting/Activated)**		
Total Cells	2,251 / 2,512	1,918 / 2,191
T cells	1,826 / 2,080	1,304 / 1,455
T cell fraction	0.811 / 0.828	0.680 / 0.664
Average Transcript Molecules Detected (T cells)	3,666 / 4,359	2,272 / 3,690
**Lymph Node (Resting/Activated)**		
Total Cells	4,896 / 4,649	3,395 / 5,820
T cells	4,194 / 4,186	2,992 / 5,190
T cell fraction	0.857 / 0.900	0.881 / 0.892
Average Transcript Molecules Detected (T cells)	3,126 / 2,888	3,320 / 3,789

	Blood Donor A	Blood Donor B
**Blood (Resting/Activated)**		
Total Cells	4,872 / 5,411	4,777 / 4,775
T cells	4,282 / 4,911	4,196 / 4,236
T cell fraction	0.879 / 0.908	0.878 / 0.887
Average Transcript Molecules Detected (T cells)	4,131 / 6,082	4,256 / 6,342

Supplementary Table 2: scRNA-seq cell numbers, T cell purity, and transcripts detected.
