# Supplementary material for: Single-cell transcriptomics of human T cells reveals tissue and activation signatures in health and disease
Source: Nat Commun. 2019 Oct 17;10:4706. doi: 10.1038/s41467-019-12464-3 (PMC6797728; doi:10.1038/s41467-019-12464-3)
Supplement: Supplementary file 8 — Reporting Summary [file 41467_2019_12464_MOESM8_ESM.pdf]

natureresearch

Corresponding author(s): D.L. Farber and P.A. Sims

Last updated by author(s): Aug 23, 2019

Reporting Summary

Nature Research wishes to improve the reproducibility of the work that we publish. This form provides structure for consistency and transparency in reporting. For further information on Nature Research policies, see Authors & Referees and the Editorial Policy Checklist.

Statistics

For all statistical analyses, confirm that the following items are present in the figure legend, table legend, main text, or Methods section.

n/a	Confirmed	
☐	☒	The exact sample size (*n*) for each experimental group/condition, given as a discrete number and unit of measurement
☐	☒	A statement on whether measurements were taken from distinct samples or whether the same sample was measured repeatedly
☐	☒	The statistical test(s) used AND whether they are one- or two-sided<br>*Only common tests should be described solely by name; describe more complex techniques in the Methods section.*
☒	☐	A description of all covariates tested
☐	☒	A description of any assumptions or corrections, such as tests of normality and adjustment for multiple comparisons
☐	☒	A full description of the statistical parameters including central tendency (e.g. means) or other basic estimates (e.g. regression coefficient) AND variation (e.g. standard deviation) or associated estimates of uncertainty (e.g. confidence intervals)
☐	☒	For null hypothesis testing, the test statistic (e.g. *F*, *t*, *r*) with confidence intervals, effect sizes, degrees of freedom and *P* value noted<br>*Give P values as exact values whenever suitable.*
☒	☐	For Bayesian analysis, information on the choice of priors and Markov chain Monte Carlo settings
☒	☐	For hierarchical and complex designs, identification of the appropriate level for tests and full reporting of outcomes
☐	☒	Estimates of effect sizes (e.g. Cohen's *d*, Pearson's *r*), indicating how they were calculated

Our web collection on statistics for biologists contains articles on many of the points above.

Software and code

Policy information about availability of computer code

Data collection	FACS Diva v8
Data analysis	FCS Express 6, GraphPad Prism 8, Expression Suite 1.1, STAR (https://github.com/alexdobin/STAR), Phenograph (https://github.com/jacoblevine/PhenoGraph), scran (https://bioconductor.org/packages/release/bioc/html/scran.html), UMAP (https://github.com/lmcinnes/umap), DMAPS (https://github.com/hsidky/dmaps), scHPF (https://github.com/simslab/scHPF), TensorFlow (https://www.tensorflow.org/), Samtools v1.3 (http://samtools.sourceforge.net/), Cython (https://cython.org/), Python 3.6 (https://www.python.org/downloads/release/python-360/), GSEA (http://software.broadinstitute.org/gsea/index.jsp), scmap (https://github.com/hemberg-lab/scmap).

For manuscripts utilizing custom algorithms or software that are central to the research but not yet described in published literature, software must be made available to editors/reviewers. We strongly encourage code deposition in a community repository (e.g. GitHub). See the Nature Research guidelines for submitting code & software for further information.

Data

Policy information about availability of data

All manuscripts must include a data availability statement. This statement should provide the following information, where applicable:
- Accession codes, unique identifiers, or web links for publicly available datasets
- A list of figures that have associated raw data
- A description of any restrictions on data availability

scRNAseq data have been uploaded to GEO, accession number GSE126030. All figures have associated raw data. No restrictions on data availability

Field-specific reporting

Please select the one below that is the best fit for your research. If you are not sure, read the appropriate sections before making your selection.

☒ Life sciences ☐ Behavioural & social sciences ☐ Ecological, evolutionary & environmental sciences

For a reference copy of the document with all sections, see nature.com/documents/nr-reporting-summary-flat.pdf

Life sciences study design

All studies must disclose on these points even when the disclosure is negative.

Sample size	The data obtained were used to define qualitative profiles, and not quantitative measurements. Therefore, no power analysis was done to calculate sample size.
Data exclusions	No data were excluded in this study.
Replication	Experimental findings were reliably reproduced with different donors across multiple experiments.
Randomization	Groups of individuals were not compared, therefore no randomization was necessary.
Blinding	Blinding was not relevant as all individuals were compared to each other.

Reporting for specific materials, systems and methods

We require information from authors about some types of materials, experimental systems and methods used in many studies. Here, indicate whether each material, system or method listed is relevant to your study. If you are not sure if a list item applies to your research, read the appropriate section before selecting a response.

Materials & experimental systems

n/a	Involved in the study	
☐	☒	Antibodies
☒	☐	Eukaryotic cell lines
☒	☐	Palaeontology
☒	☐	Animals and other organisms
☐	☒	Human research participants
☒	☐	Clinical data

Methods

n/a	Involved in the study	
☒	☐	ChIP-seq
☐	☒	Flow cytometry
☒	☐	MRI-based neuroimaging

Antibodies

Antibodies used

Anti-human CD3-BUV395, BD Biosciences, clone UCHT1 Cat#563546
Anti-human CD4-BUV737, BD Biosciences, clone SK3, Cat#612748
Anti-human CD8-BUV496, BD Biosciences, clone RPA-T8, Cat#564804
Anti-human CCR7-AlexaFluor488, BioLegend, clone G043H7, Cat#353206
Anti-human CD45RA-BV605, BioLegend, clone HI100, Cat#304134
Anti-human CD25-APC, BioLegend, clone BC96, Cat#302610
Anti-human CD127-BV510, BioLegend, clone A019D5, Cat#351332
Anti-human CD69-BV711, BioLegend, clone FN50, Cat#563836
Anti-human CD103-PECy7, BioLend, clone Ber-ACT8, Cat#350212
Anti-human CD45-AlexaFluor700, BioLegend, clone HI30, Cat#304024
Anti-human NME1-PE, Sino Biological, clone #01, Cat# 11615-MM01-P
Human Type 1 IFN Neutralizing Antibody Mixture, PBL Assay Science, Cat# 39000-1
Anti-human IFN-g, R&D Systems, clone 25718, Cat# MAB285-SP
Anti-human IFN-gR1, R&D Systems, clone 92101, Cat# MAB6731-SP

Validation

All flow cytometry antibodies were titrated and extensively validated using human blood and tissue samples. Functional antibodies were validated using positive controls in each experiment (data shown).

Human research participants

Policy information about studies involving human research participants

Population characteristics

Tissue donor characteristics described in Supplementary Table 1. Peripheral blood was obtained from consenting healthy adults, both male and female, aged 30-55.

Recruitment	Healthy adult peripheral blood donors were recruited from Columbia University Medical Center.
Ethics oversight	Columbia IRB AAAP8763

Note that full information on the approval of the study protocol must also be provided in the manuscript.

Flow Cytometry

Plots

Confirm that:

☒ The axis labels state the marker and fluorochrome used (e.g. CD4-FITC).

☒ The axis scales are clearly visible. Include numbers along axes only for bottom left plot of group (a 'group' is an analysis of identical markers).

☒ All plots are contour plots with outliers or pseudocolor plots.

☒ A numerical value for number of cells or percentage (with statistics) is provided.

Methodology

Sample preparation	Lymph Nodes and Lungs were harvested, mechanically processed by a GentleMACS tissue dissociator, enzymatically digested with collagenase D for 1 hour, and centrifuged on a density gradient. Bone marrow and blood mononuclear cells were isolated by density centrifugation. Surface antigens were stained in cold PBS+2% FCS and intracellular staining was performed with the Tonbo Foxp3/Transcription Factor Staining Buffer Kit according to manufacturer's instructions.
Instrument	BD Influx, BD LSR II
Software	FACS Diva 8, FCS Express
Cell population abundance	Up to 10x10^6 CD4+ or CD8+ T cells were sorted from the blood. Purity was typically >98%.
Gating strategy	All samples were first gated on singlets, FSClow and SSClow, CD45 positive, Viability Dye- (indicating live cells). The gating strategy for phenotyping is shown in Supplementary Figure 18a and the sorting strategy is shown in Supplementary Figure 18b.

☒ Tick this box to confirm that a figure exemplifying the gating strategy is provided in the Supplementary Information.
